# Meta-analysis of haplotype-association studies: comparison of methods and empirical evaluation of the literature

**DOI:** 10.1186/1471-2156-12-8

**Published:** 2011-01-19

**Authors:** Pantelis G Bagos

**Affiliations:** 1 Department of Computer Science and Biomedical Informatics, University of Central Greece, Papasiopoulou 2-4, Lamia 35100, Greece

## Abstract

**Background:**

Meta-analysis is a popular methodology in several fields of medical research, including genetic association studies. However, the methods used for meta-analysis of association studies that report haplotypes have not been studied in detail. In this work, methods for performing meta-analysis of haplotype association studies are summarized, compared and presented in a unified framework along with an empirical evaluation of the literature.

**Results:**

We present multivariate methods that use summary-based data as well as methods that use binary and count data in a generalized linear mixed model framework (logistic regression, multinomial regression and Poisson regression). The methods presented here avoid the inflation of the type I error rate that could be the result of the traditional approach of comparing a haplotype against the remaining ones, whereas, they can be fitted using standard software. Moreover, formal global tests are presented for assessing the statistical significance of the overall association. Although the methods presented here assume that the haplotypes are directly observed, they can be easily extended to allow for such an uncertainty by weighting the haplotypes by their probability.

**Conclusions:**

An empirical evaluation of the published literature and a comparison against the meta-analyses that use single nucleotide polymorphisms, suggests that the studies reporting meta-analysis of haplotypes contain approximately half of the included studies and produce significant results twice more often. We show that this excess of statistically significant results, stems from the sub-optimal method of analysis used and, in approximately half of the cases, the statistical significance is refuted if the data are properly re-analyzed. Illustrative examples of code are given in Stata and it is anticipated that the methods developed in this work will be widely applied in the meta-analysis of haplotype association studies.

## Background

The continuously increasing number of published gene-disease association studies made imperative the need of collecting and synthesizing the available data [1,2]. The statistical procedure with which data from multiple studies are synthesized is known as meta-analysis [3-5]. In meta-analysis, a set of original studies is synthesized and the potential heterogeneity is explored using formal statistical methods [3,4,6,7]. In the medical literature, meta-analysis was initially applied in the field of randomized clinical trials [8,9], but nowadays it is considered a valuable tool for the combination of observational studies [10], as well as for genetic association studies for which specialized methodology has been developed [5,11-18].

Most of the genetic association studies (and hence the meta-analyses derived from them) are performed using single markers, usually Single Nucleotide Polymorphisms (SNPs). However, the SNP that is under investigation is not always the true susceptibility allele. Instead, it may be a polymorphism which is in Linkage Disequilibrium (LD) with the unknown disease-causing locus [19]. In such cases, the single marker tests may be underpowered, depending on the degree of LD and the allele frequencies [20]. Haplotypes, which are the combination of closely linked alleles on a chromosome, are therefore important in the study of the genetic basis of diseases and thus, they are extensively used [21,22]. The importance of studying haplotypes ranges from elucidating the exact biological role played by neighbouring amino-acids on the protein structure, to providing information about ancient ancestral chromosome segments that harbour alleles influencing human traits [23]. Moreover, haplotype association methods are considered to be more powerful compared to single marker analyses [24,25], even though this is questioned by some researchers [26].

**Figure 1:**
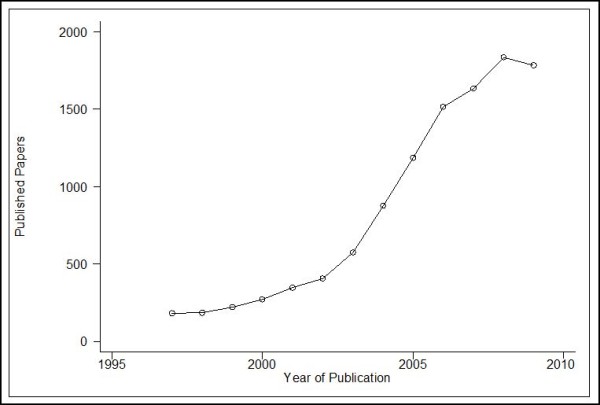
**A graphical representation of the increasing number of published haplotype-association studies**. A search was performed in Pubmed using the terms "haplotype" and "association" from 1997 to 2009. Even though the reference list may include review articles, methodological papers or even irrelevant works, the trend is obvious, especially after 2003 when the HapMap project was presented. The search was conducted during December 2009 and thus the count for 2009 may be an underestimate.

A major problem in haplotype analyses is that in order for the analysis to be performed we need to reconstruct or infer the haplotypes, usually with an approach based on missing data imputation [27-29]. This uncertainty in imputing the haplotypes poses some problems in the analysis [30] that are to be discussed later in this work. Nevertheless, studies that investigate the association of haplotypes with diseases are increasingly being published (Figure 1), with an even more increasing rate after 2003, when the HapMap project was initiated [31]. This exponential increase follows the general pattern of gene-disease association studies [1,2,32] and naturally, the obvious extension would be to use meta-analysis in order to increase the power of individual studies and to resolve the reasons of heterogeneity and inconsistency.

This work has two primary goals. First, to perform a detailed literature search and an empirical evaluation of the published studies that report meta-analyses of haplotype associations; and second, to present a concise overview of the statistical methods that could and should be used in such meta-analyses. These two important issues were not previously studied in the literature and the findings are interesting. Even though the methods presented in this work could be derived in a straightforward manner from extending previous works on multivariate meta-analysis [33-37], the majority of the published meta-analyses did not use optimal methods for analyzing the data. Moreover, in several circumstances the results of some studies are shown to be severely flawed. The manuscript is organized as follows: Initially, the commonly used methods for haplotype analysis for a single study are reviewed in order to establish notation. Afterwards, the methods of meta-analysis are presented. In particular, we present the standard method of univariate meta-analysis and its limitations, which leads to a more powerful multivariate approach based on summary-data. Accordingly, a general framework based on generalized linear mixed models (GLMMs) is presented and the approaches based on logistic regression, multinomial logistic regression and Poisson regression are discussed. We also discuss continuous traits and details of the implementation of the models. Finally, we present the results of the empirical evaluation of the literature and compare the results reported in these analyses with the ones obtained using the methods developed here.

## Methods

### Methods for haplotype association

Let's assume we have *n *biallelic markers that form a haplotype. If the alleles in position *m *(*m *= 1, 2... *n*) are denoted by *A*^*m *^and *B*^*m *^the possible haplotypes would be *r *= 2^*n*^. In a case-control study, a cross-tabulation of haplotypes by disease status, that ignores the individuals and counts only the total number of haplotypes observed in the analysis, would result in data arranged in the form of a 2 × *r *contingency table (Table 1). This cross-tabulation is somehow simplistic since it assumes a multiplicative (co-dominant) model of inheritance [38]. However, it is the most commonly reported form of haplotype data and thus, it is more suitable for meta-analysis of published studies as we will discuss later. Assuming a binomial sampling scheme where fixed numbers of cases and controls are sampled independently, we can model the structure of the table using logistic regression methods where the status (case/control) is the dependent variable and the haplotypes are treated as covariates. This corresponds to the so-called "prospective likelihood", the likelihood based on the probability of the disease given the exposure. Thus, we denote by *π*_*j *_= *P*(*y*_*j *_= 1) the underlying risk (i.e. the probability of being a case) of a person carrying a single copy of the *j*^*th *^haplotype. A reasonable choice would be to consider the most common haplotype (i.e. *h*_1_) as the reference category and create *r*-1 dummy variables taking values *z*_*j *_= 1 for haplotype *j *and 0 otherwise. This model can be formulated as:

**Table 1:** Cross-tabulation of haplotypes by disease status

**Haplotype (*z***_***j***_**)**	Cases (*y *= 1)	Controls (*y *= 0)
1	89	183
2	14	26
3	24	22
4	3	3

The haplotype data obtained in a case-control study on 182 caucasian women concerning the association of p53 haplotypes with breast cancer [108]. The data are presented in the form described by Wallenstein and coworkers [38].

(1)$$\text{logit}\left(\pi_{j}\right)=\text{logit}\left[P\left(y_{j}=1|j\right)\right]=\beta_{0}+\sum_{j=2}^{r}\beta_{j}z_{j}$$

This model was proposed initially by Wallenstein and co-workers and as we already mentioned, assumes a multiplicative genetic model of inheritance [38]. Moreover, the haplotypes are assumed known quantities, which may not always be the case (see below).

Alternatively, assuming a multinomial sampling scheme where the total sample size is considered fixed, a multinomial logistic regression model would be appropriate, where the different haplotypes would be the dependent variables. This corresponds to the well-known "retrospective likelihood" (i.e. the likelihood based on the probability of exposure given disease status) applicable in case control studies. In this case, the haplotypes are treated as dependent variables and the case/control status as the predictor in a multinomial (polytomous or polychotomous) logistic regression [39]:

(2) $$p_{j}=P\left(j|y_{j}\right)\text{= }\frac{\text{exp}\left(\alpha_{j}+\beta_{j}y_{j}\right)}{\sum_{j=1}^{r}\text{exp}\left(\alpha_{j}+\beta_{j}y_{j}\right)}$$

By observing that the linear predictor becomes:

(3) $$U_{j}=\log\left(\frac{p_{j}}{p_{1}}\right)=a_{j}+\beta_{j}y_{j},j=1,2,...,r$$

it is easy to understand that the *β*_*j *_coefficients obtained by fitting the model are estimates of the log-Odds Ratios (i.e. for comparing *h*_*j *_vs. *h*_1_) in equivalence to the respective coefficients of the model in Eq. (1). Obviously, *β*_1 _= 0 for identifiability since haplotype *j *= 1 (i.e. *h*_1_) is used as the reference category. The particular model was first used for haplotype analysis by Chen and Kao [40].

Lastly, assuming that the observed counts are realizations of a Poisson random variable, one can fit log-linear models (Poisson regression), where the dependent variable is the counts and thus, the studies, the type of haplotypes and the case/control status are treated as independent variables. Log-linear models are widely used for haplotype analysis, for instance, for detecting LD [41,42] and for haplotype-disease association [43,44]. This model can be formulated in terms of a Poisson regression model in the context of generalized linear models, as:

(4) $$\log\left(n_{j}\right)=\mu_{0}+\beta_{0}y_{j}+\sum_{j=2}^{r}a_{j}z_{j}+\sum_{j=2}^{r}\beta_{j}z_{j}y_{j}$$

This is the standard saturated model for describing the 2 × *r *contingency table of haplotypes by disease. The *β*_*j*_'s are the coefficients that correspond to the haplotype by disease interaction and are equivalent to those obtained by fitting the models in Eq. (1) and (2). It is easily verified that the coefficients *α*'s and *β*'s are identical across the three models. The overall hypothesis for association (**β **= **0**) can be tested by performing a multivariate Wald test using the estimated covariance matrix, cov(**β**). Then, the test statistic (score) *U *= **β'**cov(**β**)^-1^**β**, will have asymptotically a *χ*^2 ^distribution on *r*-1 degrees of freedom. Alternatively, a likelihood ratio test comparing the saturated model against the model with no interaction can be performed. Similar tests can be performed for the models in Eq. (1) and (2).

Whatever the assumed sampling scheme that gave rise to the data of Table 1 may be, it is well known that the results of fitting each one of the three models are nearly identical [45]. For instance, it has been shown that maximum likelihood estimates obtained from the "retrospective" likelihood are the same as those obtained from the "prospective" likelihood [46,47]. The equivalence of logistic regression and Poisson modelling has been also exploited in the past for deriving methods for detecting gene-environment interactions [48].

The methods discussed above are simple applications of the generalized linear model extending the analysis of single markers to haplotypes and assume that, i) the haplotype risk follows a multiplicative model of inheritance, ii), the haplotype phase is known and, iii) the population is in Hardy-Weinberg Equilibrium (HWE). The genetic model of inheritance can be handled simply by using in the analysis the so-called haplo-genotypes or diplotypes, instead of the genotypes. This is easily performed with all the previously presented methods by using the pairwise combinations of haplotypes (*h*_1_*h*_1_, *h*_1_*h*_2 _and so on). In case-control association studies, however, with the exception of some cases where direct genotyping of the haplotypes is applicable (i.e. [38]), the haplotypes (and the haplo-genotypes) are usually not known, but are inferred from the data using statistical methods for missing data, usually with an EM or EM-like algorithm [27-29]. Thus, treating them as known quantities has been shown to be problematic [30]. More advanced methods have been developed in order to account for these limitations, for instance weighting the haplotypes by their probability [49,50]. Score methods based on the prospective likelihood [51] or the retrospective likelihood [52], have also been developed, as well as methods for allowing for gene-environment interaction [53]. A comparison of methods has shown that the approaches are roughly comparable when the haplotype effect on disease odds follows a multiplicative model. However, for dominant and recessive models, the retrospective-likelihood method has increased efficiency with respect to the prospective methods [54]. Graphical models have been proposed by Thomas [55] and log-linear models by Baker [56]. Lin and co-workers extended the previously presented methods by including various sampling schemes in a unified framework [57].

Even though a large body of the genetic epidemiology literature is dedicated to such methods, their application in meta-analysis is problematic since in most cases the original data are not available to the analyst. Thus, in the following sections where the methods for meta-analysis are summarized we also assume that the haplotypes are known. An extension when the posterior probabilities of haplotypes are given from the output of the haplotype inference software would then be straightforward.

### Methods for meta-analysis of haplotype association

In this section the methods for meta-analysis are presented. Initially we will discuss simple methods using summary data, whereas in the next sub-section more advanced methods that use generalized linear models on grouped or Individual Patients Data (IPD) are presented.

### Meta-analysis using summary-data

A commonly used approach that is based on traditional methods and uses solely summary data is to consider separately the effect of the *j*^th ^haplotype against the *j*-1 remaining ones. That is, for each study *i *(*i *= 1,2,...,*k*) we will compute a log-Odds Ratio (logOR):

(5) $$y_{i}=\log OR_{i}=\log\left(\frac{n_{ij1}n_{ic0}}{n_{ij0}n_{ic1}}\right)$$

with an asymptotic variance given by:

(6) $$\mathrm{var}\left(y_{i}\right)=s_{i}^{2}=\frac{1}{n_{ij1}}+\frac{1}{n_{ic0}}+\frac{1}{n_{ij0}}+\frac{1}{n_{ic1}}$$

In this notation, *n*_*ic*__0 _an *n*_*ic*__1_, are the counts of the remaining haplotypes (excluding haplotype *j*) for controls and cases of the *i*^th ^study respectively, given by:

(7) $$\begin{array}{l} n_{ic0}=\sum_{j'=1}^{r}n_{ij'0}-n_{ij0}=\sum_{j'=1|j'\neq j}^{r}n_{ij'0}\\ n_{ic1}=\sum_{j'=1}^{r}n_{ij'1}-n_{ij1}=\sum_{j'=1|j'\neq j}^{r}n_{ij'1} \end{array}$$

In a standard univariate random-effects model we assume that the logarithm of the OR of each study *i*, is distributed normally as:

(8) $$y_{i}\sim N\left(\beta ,s_{i}^{2}+\tau^{2}\right)$$

Thus, the combined logarithm of the Odds Ratio (log*OR*) would be given by:

(9) $$\overset{\wedge }{\beta }=\sum_{i=1}^{k}w_{i}\beta_{i}/\sum_{i=1}^{k}w_{i},\text{ with }w_{i}=1/\left(s_{i}^{2}+\tau^{2}\right)$$

The between-studies variance (*τ*^2^), could be easily computed by the non-iterative method of moments proposed by Dersimonian and Laird [58], even though there are several alternatives that use iterative procedures (i.e. Maximum Likelihood (ML) or Restricted Maximum Likelihood (REML) [33]). Apparently, by setting *τ*^2 ^= 0 in Eq. (9) corresponds to the well known fixed-effects estimator with inverse variance weights.

The particular approach is very easily implemented, intuitive and it can be performed in a standard univariate meta-analysis framework. In the results section we will see that several already published meta-analyses used this method. However, the method has some drawbacks. The most important is that it is prone to an increased type I error rate due to multiple comparisons. Multiple comparisons constitute an important problem in haplotype analysis, especially as the number of haplotypes increases [59,60]. The model implied by Eqs. (5) - (8), is conceptually similar to collapsing the genotypes in a single-marker analysis, an approach that has been shown to increase the power as well the type I error rate [61]. Thus, the particular approach can be justified, only when there is strong prior knowledge concerning a particular haplotype and this haplotype is the only one that is being tested.

To overcome the multiple comparisons problem, a straightforward alternative would be to extend the model in a multivariate framework modelling simultaneously the logORs derived from comparing haplotypes *j *= 2,3,...,*r *against a reference haplotype (*j *= 1). Following the general framework for multivariate meta-analysis [37,62], we denote by **y**_*i *_the vector containing the *r*-1 different estimates, and by **β**, the vector of the overall means given by:

(10) $${\text{y}}_{i}=\left(\begin{array}{l} y_{2i}\\ y_{3i}\\ ...\\ y_{ri} \end{array}\right),\text{ and }\beta =\left(\begin{array}{l} \beta_{2}\\ \beta_{3}\\ ...\\ \beta_{r} \end{array}\right)$$

These logORs similarly to Eq. (5) will be given by:

(11) $$y_{ji}=\log OR_{ji}=\log\left(\frac{n_{ij1}n_{i10}}{n_{ij0}n_{i11}}\right)$$

with an asymptotic variance given by:

(12) $$\mathrm{var}\left(y_{ji}\right)=s_{ji}^{2}=\frac{1}{n_{ij1}}+\frac{1}{n_{i10}}+\frac{1}{n_{ij0}}+\frac{1}{n_{i11}}$$

In the multivariate random-effects meta-analysis, we assume that **y**_*i *_is distributed following a multivariate normal distribution around the true means **β**, according to the marginal model:

(13) $${\text{y}}_{i}\sim MVN\left(\beta ,\Sigma +{\text{C}}_{i}\right)$$

In the above model, we denote by **C**_*i *_the within-studies covariance matrix:

(14) $${\text{C}}_{i}=\left(\begin{array}{cccc} s_{2i}^{2} & \rho_{W23}s_{2i}s_{3i} & ... & \rho_{W2r}s_{2i}s_{ri}\\ \rho_{W23}s_{2i}s_{3i} & s_{3i}^{2} & ... & \rho_{W3r}s_{3i}s_{ri}\\ ... & ... & ... & ...\\ \rho_{W2r}s_{2i}s_{ri} & \rho_{W3r}s_{3i}s_{ri} & ... & s_{ri}^{2} \end{array}\right)$$

and by **Σ **the between-studies covariance matrix, given by:

(15) $$\Sigma =\left(\begin{array}{cccc} \tau_{2}^{2} & \rho_{B23}\tau_{2}\tau_{3} & ... & \rho_{Br2}\tau_{2}\tau_{r}\\ \rho_{B23}\tau_{2}\tau_{3} & \tau_{3}^{2} & ... & \rho_{Br3}\tau_{3}\tau_{r}\\ ... & ... & ... & ...\\ \rho_{Br2}\tau_{2}\tau_{r} & \rho_{Br3}\tau_{3}\tau_{r} & ... & \tau_{r}^{2} \end{array}\right)$$

The diagonal elements of **C**_*i *_are the study-specific estimates of the variance that are assumed known, whereas the off-diagonal elements correspond to the pairwise within-studies covariances, for instance *ρ*_*w*__23_s_2__*i*_s_3__*i*_=cov(*y*_2__*i*_, *y*_3__*i*_). Since the logORs derived for each haplotype are compared against the same reference category, their pairwise covariances will be given [12], by:

(16) $$\mathrm{cov}\left(y_{ji},y_{j'i}\right)=\frac{1}{n_{i10}}+\frac{1}{n_{i11}},\forall j,j'=2,3,...r,j\neq j'$$

We should mention that from standard normal theory it is known that the multivariate test for **β = 0**, based on **β'**cov(**β**)^-1^**β**, could yield significant results even if all the *r*-1 univariate Wald tests are non-significant. Thus, the multivariate test should be performed initially and only if a significant result is found we can proceed by collapsing the haplotypes and perform a standard univariate meta-analysis.

The model can be fitted in any statistical package capable of fitting random-effects weighted regression models with an arbitrary covariance matrix, such as SAS (using PROC MIXED or PROC NLMIXED), R (using lme) or Stata (using mvmeta). In this work, we used mvmeta which performs inferences based on either Maximum Likelihood (ML) or Restricted Maximum Likelihood (REML), by direct maximization of the approximate likelihood using a Newton-Raphson algorithm [63]. Alternatively, mvmeta can also implement the multivariate version of the DerSimonian and Laird's method of moments [64]. The last option, being non-iterative, is very attractive in case of large number of haplotypes and/or large number of studies. A major disadvantage of the methods proposed in this section is the assumptions of normality that are employed and the need for correction when there are rare haplotypes (i.e. adding a pseudocount of 0.5 to the haplotypes with zero counts). These limitations are surpassed by using the methods discussed in the next section.

### Meta-analysis using binary data

In this section, methods that use directly the binary nature of the data, within a generalized linear mixed model (GLMM) are presented. These methods are usually termed IPD methods [33-37] although in many real-life applications, individual data may not be literally available. Instead, extending the models described for a single study, only summary counts of individuals carrying the respective haplotypes will normally be used.

#### Logistic regression

Using the prospective likelihood we can extend the logistic regression model of Eq. (1) in order to incorporate study specific effects and perform a stratified analysis (fixed effects meta-analysis). To do so, we need to introduce *k*-1 dummy variables *d*_*i *_(taking values equal to zero or one) with coefficients *β*_0__*i *_that are indicators of the study-specific fixed-effects. Thus, the model is a straightforward extension to the model described previously for meta-analysis of genetic association studies for single nucleotide polymorphisms [16] and is formulated as:

(17) $$\begin{array}{l} \text{logit}\left(\pi_{ij}\right)=\text{logit}\left[P\left(y_{ij}=1|j\right)\right]\\ \;=\beta_{0}+\beta_{0i}d_{i}+\sum_{j=2}^{r}\beta_{j}z_{j} \end{array}$$

Here, the *β*_*j *_obtained by fitting the model are the overall estimates of the logORs (i.e. for comparing *h*_*j *_vs. *h*_1_). An overall test for the association of haplotypes with disease can be performed if we denote by **β **the vector of the estimated coefficients and by cov(**β) **its estimated variance-covariance matrix. Then, the test statistic *U *= **β**'cov(**β**)^-1^**β **will have asymptotically a *χ*^*2 *^distribution (*U*~*χ*^2^_*r*__-1_) [65]. The particular model has been used in several meta-analyses of haplotype association studies [66-69] (see in the results section, the empirical evaluation of the literature). This fixed effects model assumes homogeneity of ORs between studies. This assumption can be tested by adding the interaction between the study effect and the haplotypes into the model::

(18) $$\begin{array}{l} \text{logit}\left(\pi_{ij}\right)=\text{logit}\left[P\left(y_{ij}=1|j\right)\right]\\ \;=\beta_{0}+\beta_{0i}d_{i}+\sum_{j=2}^{r}\beta_{j}z_{j}\\ \;+\sum_{j=2}^{r}\sum_{i=2}^{k}\gamma_{ij}d_{i}z_{j} \end{array}$$

This is the analogue to the Cochran's test for heterogeneity in the univariate meta-analysis. The hypothesis can be tested by performing a multivariate Wald test, where the null hypothesis is:

$$H_{0}:\gamma =\text{0 }\left(\gamma_{ij}=0,\forall i=2,3,...,k;j=2,3,...,r\right)$$

The test statistic can be constructed analogously to the one used for **β**. If we denote by **γ **the vector of the estimated coefficients, by **V **the estimated variance-covariance matrix and by **Rγ = r **the vector of the (*r*-1)(*k*-1) linear hypotheses, then the statistic:

(19) $$W={\left(\text{R}\gamma -\text{r}\right)}^{\prime }{\left(\text{R}\text{V}{\text{R}}^{\prime }\right)}^{-1}\left(\text{R}\gamma -\text{r}\right)=\gamma^{\prime }\mathrm{cov}{\left(\gamma \right)}^{-1}\gamma$$

will have asymptotically a *χ*^*2 *^distribution [65]

(20) $$W\sim \chi_{\left(r-1\right)\left(k-1\right)}^{2}$$

Moreover, the value of *W *could be used in order to calculate a modified version of the overall inconsistency index *I*^*2 *^[70]:

(21) $$I^{2}=\max\left\{0,\frac{W-\left(r-1\right)\left(k-1\right)}{W}\right\}$$

This measure is quite useful, since it enables us to summarize the overall heterogeneity, instead of having to look at multiple indices of heterogeneity arising from multiple haplotype contrasts.

In order to account for an additive component of heterogeneity and perform a random-effects logistic regression allowing the haplotype effects to vary between studies, the most suitable way is to introduce a set of study-specific random coefficients, representing the deviation of study's true effect from the overall mean effect for each haplotype. Thus, the model becomes:

(22) $$\begin{array}{l} \text{logit}\left(\pi_{ij}\right)=\text{logit}\left[P\left(y_{ij}=1|j\right)\right]\\ \;=\beta_{0}+\beta_{0i}d_{i}+\sum_{j=2}^{r}\left(\beta_{j}+\beta_{ji}\right)z_{j} \end{array}$$

In this model, the random terms **β**_*i *_are distributed as:

(23) $$\beta_{i}\sim MVN\left(\text{0},\Sigma \right)$$

where

(24) $$\begin{array}{l} \beta_{i}=\left(\begin{array}{c} \beta_{2i}\\ \beta_{3i}\\ ...\\ \beta_{ri} \end{array}\right)\\ \Sigma =\left(\begin{array}{cccc} \tau_{2}^{2} & \rho_{B23}\tau_{2}\tau_{3} & ... & \rho_{Br2}\tau_{2}\tau_{r}\\ \rho_{B23}\tau_{2}\tau_{3} & \tau_{3}^{2} & ... & \rho_{Br3}\tau_{3}\tau_{r}\\ ... & ... & ... & ...\\ \rho_{Br2}\tau_{2}\tau_{r} & \rho_{Br3}\tau_{3}\tau_{r} & ... & \tau_{r}^{2} \end{array}\right) \end{array}$$

The between studies variances and covariances have the same interpretation as the ones obtained by the summary-data methods of Eq. (13) and (15).

#### Multinomial logistic regression

Alternatively, the model may be parameterized assuming a multinomial sampling scheme utilizing the retrospective likelihood. In this case, an extension of the model of Eq. (2), which incorporates fixed-study effects, would be:

(25) $$p_{ij}=P\left(j|y_{ij}\right)=\frac{\text{exp}\left(\alpha_{j}+\alpha_{ij}d_{i}+\beta_{j}y_{ij}\right)}{\sum_{j=1}^{r}\text{exp}\left(\alpha_{j}+\alpha_{ij}d_{i}+\beta_{j}y_{ij}\right)}$$

The linear predictor in the above model becomes:

(26) $$U_{ij}=\log\left(\frac{p_{ij}}{p_{i1}}\right)=\alpha_{j}+\alpha_{ij}d_{i}+\beta_{j}y_{ij},j=1,2,...,r$$

Similar to the model based on prospective likelihood, the variables *d*_*i *_are indicators of the study-specific fixed-effects. An overall test for the association of haplotypes with disease (**β = 0**) can be performed similarly to the logistic regression model (*U*). Introducing the study by disease interaction terms can form a test for homogeneity of ORs across the k studies:

(27) $$\begin{array}{l} U_{ij}=\log\left(\frac{p_{ij}}{p_{i1}}\right)=\alpha_{j}+\alpha_{ij}d_{i}+\beta_{j}y_{ij}+\sum_{i=2}^{k}\sum_{j=2}^{r}\gamma_{ij}d_{i}y_{ij}\\ \;j=1,2,...,r \end{array}$$

The statistics for heterogeneity (*W*) as well as the *I*^2 ^index derived from it are identical to the one presented in Eq. (19) - (21).

A random-effects extension to the model can be formulated if in the above model, we introduce a haplotype-specific random coefficient *β*_*ij *_(for haplotypes *j *= 2,3, ...,*r*), in which case the linear predictor becomes [71]:

(28) $$\begin{array}{l} U_{ij}=\log\left(\frac{p_{ij}}{p_{i1}}\right)=\alpha_{j}+\alpha_{ij}d_{i}+\beta_{j}y_{ij}+\beta_{ij}y_{ij}\\ \;j=2,3,...,r \end{array}$$

and the model is completely specified as a random effects multivariate meta-analysis, with random terms **β**_*i *_distributed similarly as **β**_*i*_~*MVN*(**0,Σ**). The interpretation of the variances and covariances of the random terms is identical to the ones presented in Eq. (13). A version of this model has been used previously for meta-analysis of genetic association studies involving single nucleotide polymorphisms [12], but according to the author's knowledge it has never been used for meta-analysis of haplotypes.

#### Poisson regression

Lastly, we can extend the log-linear model of Eq. (4) in order to perform a fixed effects meta-analysis allowing for the study-specific effects. The major difference compared to the previous approaches lies in the structure of the log-linear model and the interpretation of the main effects and interactions. Having in mind that we want to model a 2 × *r *× *k *contingency table, the appropriate choice would be to include in the model of Eq. (4) the study specific main effects as well as the two-way interactions (study x disease and study x haplotype). Thus, we would have a model containing all the main effects as well as all the two-way interactions, a model known as the "*no three-factor interaction model*" [45]:

(29) $$\begin{array}{l} \log\left(n_{ij}\right)=\mu_{0}+\mu_{i}d_{i}+\beta_{0}y_{ij}\\ \;+\sum_{j=2}^{r}a_{j}z_{j}+\sum_{j=2}^{r}\beta_{j}z_{j}y_{ij}\\ \;+\sum_{j=2}^{r}\sum_{i=2}^{k}a_{ij}z_{j}d_{i}+\sum_{i=2}^{k}\beta_{0i}y_{ij}d_{i} \end{array}$$

In this model, the coefficients *α*_*j*_, *α*_*ij*_, *β*_0_, *β*_0__*j *_and *β*_*j *_correspond to the ones obtained by fitting the models in Eq. (17) and Eq. (15). The overall test for the association of the haplotypes with the disease (**β **= **0**), is known in the context of log-linear models as the test of "*partial association*" [72,73]. The model in Eq. (29), assumes homogeneity of ORs across studies. Thus, in order to test this assumption we need to include additional terms for the three-way interaction (study x disease x haplotype). This is accomplished by fitting the saturated model:

(30) $$\begin{array}{l} \log\left(n_{ij}\right)=\mu_{0}+\mu_{i}d_{i}+\beta_{0}y_{ij}\\ \;+\sum_{j=2}^{r}a_{j}z_{j}+\sum_{j=2}^{r}\beta_{j}z_{j}y_{ij}\\ \;+\sum_{j=2}^{r}\sum_{i=2}^{k}a_{ij}z_{j}d_{i}+\sum_{i=2}^{k}\beta_{0i}y_{ij}d_{i}\\ \;+\sum_{j=2}^{r}\sum_{i=2}^{k}\gamma_{ij}z_{j}d_{i}y_{ij} \end{array}$$

The test with the null hypothesis *Η*_0_: ** γ = 0 **(*γ*_*ij *_= 0,*i *= 2,3,...,*k*, *j *= 2,3,...,*r*) is identical to the ones obtained by fitting the models in Eq. (18) and (27). The three-way interaction model and its interpretation in terms of testing the homogeneity of ORs has been discussed in detail in the past [45,74-76]. Log-linear models have been employed in several meta-analyses of haplotype association [77,78] (see in the results section). However, even though not described in detail, it is apparent from the results reported, that in these analyses the log-linear model was not applied in an appropriate manner. Although the authors stated that they performed stratification by study, they probably included only the main effect of the study and not the interaction terms with both haplotypes and disease. As we will see in the results section, when the correct model is applied, the originally drawn conclusions are compromised.

In analogy to models in Eq. (22) and (28), a random coefficient for the disease by haplotype interaction can be applied in order to perform a random-effects meta-analysis:

(31) $$\begin{array}{l} \log\left(n_{ij}\right)=\mu_{0}+\mu_{i}d_{i}+\beta_{0}y_{ij}\\ \;+\sum_{j=2}^{r}a_{j}z_{j}+\sum_{j=2}^{r}\left(\beta_{j}+\beta_{ji}\right)z_{j}y_{ij}\\ \;+\sum_{j=2}^{r}\sum_{i=2}^{k}a_{ij}z_{j}d_{i}+\sum_{i=2}^{k}\beta_{0i}y_{ij}d_{i} \end{array}$$

with random terms **β**_*i *_distributed similarly as **β**_*i*_~*MVN*(**0,Σ**). Similarly to the multinomial logistic regression model, the interpretation of the variances and covariances of the random terms is identical to the ones presented in Eq. (12).

### Continuous traits

The methods discussed so far assume we are dealing with a binary trait, usually in a case-control setting. However, continuous traits are not uncommon in genetic association studies and these should be easily accommodated using a linear model (linear regression). For instance, denoting by *y*_*ij *_the continuous trait for a person carrying the *j*^th ^haplotype in the *i*^th ^study, the model would be:

(32) $$y_{ij}=\beta_{0}+\beta_{0i}d_{i}+\sum_{j=2}^{r}\beta_{j}z_{j}$$

The homogeneity of haplotype effects across studies can be subsequently checked using a model with a haplotype x study interaction term:

(33) $$y_{ij}=\beta_{0}+\beta_{0i}d_{i}+\sum_{j=2}^{r}\beta_{j}z_{j}+\sum_{j=2}^{r}\sum_{i=2}^{k}\gamma_{ij}d_{i}z_{j}$$

Finally, a random effects model could be formulated using a liner mixed model:

(34) $$y_{ij}=\beta_{0}+\beta_{0i}d_{i}+\sum_{j=2}^{r}\left(\beta_{j}+\beta_{ji}\right)z_{j}$$

with random terms **β**_*i *_distributed similarly as **β**_*i*_~*MVN*(**0,Σ**). Similarly to the previously described models, the interpretation of the variances and covariances of the random terms is identical to the ones presented in Eq. (12). In case where individual data are not available, the above models could be easily fitted using summary data (mean values and standard deviations) per haplotype.

### Implementation

The models presented in this section can be easily fitted in Stata using gllamm, or in SAS using PROC NLMIXED. These models are expected to perform better compared to the models presented in the previous section, in case the normality assumption for logORs does not hold. Furthermore, a major advantage of these models is that they can directly be used for pooled meta-analyses performed under large collaborative projects. This is why these models are usually termed Individual Patients Data (IPD) methods [36]. However, a disadvantage is that these methods are computational intensive, especially when the number of haplotypes is large.

A sometimes useful simplification can be made in Eq. (15) if we assume that the between-studies variances are equal [34]. In such case by letting *τ = τ*_2 _= *τ*_*3 *_= ... = *τ*_*r*_, **Σ **reduces to:

(35) $$\Sigma =\left(\begin{array}{cccc} \tau^{2} & \rho \tau^{2} & ... & \rho \tau^{2}\\ \rho \tau^{2} & \tau^{2} & ... & \rho \tau^{2}\\ ... & ... & ... & ...\\ \rho \tau^{2} & \rho \tau^{2} & ... & \tau^{2} \end{array}\right)$$

Another approximation would be to impose a single between studies correlation, but allow for different between-studies variances [79]:

(36) $$\Sigma =\left(\begin{array}{cccc} \tau_{2}^{2} & \rho \tau_{2}\tau_{3} & ... & \rho \tau_{2}\tau_{r}\\ \rho \tau_{2}\tau_{3} & \tau_{3}^{2} & ... & \rho \tau_{3}\tau_{r}\\ ... & ... & ... & ...\\ \rho \tau_{2}\tau_{r} & \rho \tau_{3}\tau_{r} & ... & \tau_{r}^{2} \end{array}\right)$$

In this work however, we chose to use a different approximation that can be obtained if the number of random effects is reduced by decomposing the random terms using factor loadings such as: *τ*_2_^2 ^= *λ*_2_^2^*τ*^2^, *τ*_3_^2 ^= *λ*_3_^2^*τ*^2^, ..., *τ*_*r*_^2 ^= *λ*_*r*_^2^*τ*^2^, and letting *λ*_2 _= 1 for identification. Thus, the covariance matrix becomes now:

(37) $$\Sigma =\left(\begin{array}{cccc} \tau^{2} & \lambda_{3}\tau^{2} & ... & \lambda_{r}\tau^{2}\\ \lambda_{3}\tau^{2} & \lambda_{3}^{2}\tau^{2} & ... & \lambda_{3}\lambda_{r}\tau^{2}\\ ... & ... & ... & ...\\ \lambda_{r}\tau^{2} & \lambda_{3}\lambda_{r}\tau^{2} & ... & \lambda_{r}^{2}\tau^{2} \end{array}\right)$$

The particular approximation is conceptually similar to the one used previously for the so-called "genetic model-free approach" for meta-analysis of genetic association studies [14,80], even though the motivation was different. The model imposes a single between studies variance *τ*^2 ^thus, it is much faster since the factor loadings *λ*_*j *_with *j *= 3,4,...,*r *are treated as fixed-effects parameters. By observing also the off-diagonal elements of the covariance matrix in Eq. (37), we can see that the model restricts the between-studies correlations (*ρ*_*Bjj'*_) to be equal to ±1 (depending on the sign of *λ*_*j*_*λ*_*j'*_). Nevertheless, the between-studies correlations are usually poorly estimated especially when the number of studies is small (<20) and in such cases they are usually estimated to be equal to ±1 [81,82]. Thus, the particular approach seems to be a good compromise between speed and precision and we expect to perform well. Using this approach, the computational complexity as well as the execution time is reduced drastically but the obtained estimates agree up to the fourth decimal place in most of the experiments conducted.

A final comment has to be made concerning the identifiability of the models presented in the previous sections, especially when it comes to the log-linear models which are the ones that contain the largest number of parameters. Concerning the fixed effects methods, the number of parameters of the saturated model of Eq. (30) is equal to 2*rk*, a number that is equal to the number of observations [45]. For the model of Eq. (29), the number of freely estimated parameters is equal to *rk *+ *r *+ *k*-1, which is obviously smaller than 2*rk *(since *r *> 1 and *k *> 1). The random effects model of Eq. (31) has a total number of parameters equal to *rk *+ *r *+ *k*-1 + *r*(*r-*1*)*/2 since we need to estimate additionally *r*(*r-*1*)*/2 elements of the covariance matrix (the variances and the covariances of the random effects). Thus, in order for the model to be identifiable we need to ensure that *rk *+ *r *+ *k*-1 + *r*(*r-*1*)*/2 ≤ 2*rk *which is accomplished if *k*; ≥ 1+*r*/2. Intuitively, we need a relatively larger number of studies compared to the number of haplotypes. If on the other hand, we fit the model of Eq. (31) using Eq. (35) for restricting the covariances, we only need *rk *+ *r *+ *k *parameters and when we use Eq. (36) or Eq. (37), we need to estimate *rk*+2*r *+ *k*-1 parameters, numbers which both are smaller than 2*rk*. Nevertheless, for practical applications, we will normally use the logistic regression model of Eq. (22) coupled with parameterization of Eq. (37), and thus identifiability issues will never arise in practice.

In Additional file 1, Stata programs for fitting the models developed in this section are presented. The models were fitted using the gllamm module for Stata [83,84]. gllamm uses numerical integration by adaptive quadrature in order to integrate out the latent variables and obtain the marginal log-likelihood. Afterwards, the log-likelihood is maximized by Newton-Raphson using numerical first and second derivatives.

## Results

We initially performed a literature search for identifying studies that report meta-analyses of haplotype associations. The initial search in PUBMED using the term "haplotype" combined with "meta-analysis" or "collaborative analysis" or "pooled analysis" yielded 282 studies. Of these, 35 studies could have been identified using solely the terms "collaborative analysis" or "pooled analysis" and "haplotype". After careful screening, 207 studies were excluded as irrelevant ones (they were not meta-analyses of haplotypes), 36 studies were excluded for various reasons (family based-studies, meta-analyses of SNPs with the term "haplotype" appearing in the abstract or haplotype analyses in which the term "meta-analysis" appeared in the abstract etc). Finally, we came up with 39 published papers containing data for 43 associations. Some studies reported different sets of haplotypes from the same gene (Auburn et al, 2008; Zintzaras et al, 2009), haplotypes from different genes (Thakkinstian et al, 2008), or distinct outcomes measured on different subsets of patients (Kavvoura et al, 2007) and thus, they were included twice, whereas from studies that reported different outcomes measured on the same set of individuals we kept only one. There were also some pairs of studies that evaluated the same association and from these we kept only the largest one. 10 out of the 39 published papers could have been identified using solely the terms "collaborative analysis" or "pooled analysis" coupled with the term "haplotype". The 43 studies and their characteristics are presented in Table 2.

**Table 2:** List of the 43 meta-analyses that were used in the empirical evaluation

ID	Reference	Gene/Locus	Disease/Outcome	SNPs in haplotype	Number of studies	Sample Size	Method of analysis	Data availability	Collaborative analysis	Significant results
1	[109]	DRD3	Schizophrenia	4	5	7551	1 vs. others	No	No	No
2	[98]	ITGAV	Rheumatoid Arthritis	3	3	6851	N/A	Yes	Yes	Yes
3	[110]	IL1A/IL1B/IL1RN	Osteoarthritis	7	4	2908	1 vs. others	No	Yes	Yes
4	[111]	FRZB	Osteoarthritis	2	10	12380	1 vs. others	No	Yes	No
5	[99]	CX3CR1	CAD	2	6	2912	1 vs. others	Yes	No	Yes
6	[112]	ALOX5AP	Stroke	4	5	5765	1 vs. others	No	No	No
7	[112]	ALOX5AP	Stroke	4	3	3004	1 vs. others	No	No	No
8	[113]	GNAS	Malaria	3	7	8154	1 vs. others	No	Yes	Yes
9	[113]	GNAS	Malaria	7	6	7632	1 vs. others	No	Yes	Yes
10	[114]	PDLIM5	Bipolar Disorder	2	3	1208	1 vs. others	No	No	No
11	[115]	PDE4D	Stroke	2	4	4961	1 vs. others	No	No	Yes
12	[116]	TGFB1	Renal Transplantation	2	4	438	pooled	No	No	Yes
13	[116]	IL10	Renal Transplantation	3	4	348	pooled	No	No	No
14	[117]	9p21.3	CAD	4	5	7838	1 vs. others	No	Yes	Yes
15	[118]	HLA	SLE	2	3	527	1 vs. others	No	No	Yes
16	[94]	CTLA4	Graves Disease	2	10	2564	1 vs. others	Yes	Yes	Yes
17	[94]	CTLA4	Hashimoto Thyroiditis	2	5	1210	1 vs. others	Yes	Yes	Yes
18	[119]	ENPP1	T2DM	3	3	8676	1 vs. others	No	No	No
19	[77]	MTHFR	ALL	2	4	894	Log-linear model	No	No	Yes
20	[97]	CAPN10	T2DM	3	11	5862	1 vs. others	Yes	Yes	Yes
21	[93]	ADAM33	Asthma	5	3	1899	pooled	Yes	No	No
22	[120]	NRG1	Schizophrenia	6	11	8722	1 vs. others	No	No	Yes
23	[121]	RGS4	Schizophrenia	4	8	7243	1 vs. others	No	Yes	No
24	[122]	ADRB2	Asthma	2	3	2060	N/A	No	No	Yes
25	[123]	ESR1	Fractures	3	8	14622	1 vs. others	No	Yes	Yes
26	[78]	VDR	Osteoporosis	3	4	2335	Log-linear model	Yes	No	Yes
27	[95]	ACE	Alzheimer's Disease	3	4	1619	pooled	Yes	Yes	Yes
28	[124]	IGF-I	IGF-I levels	3	3	1929	1 vs. others	No	Yes	Yes
29	[125]	TF	Stroke	2	2	818	N/A	No	Yes	No
30	[92]	FcgammaR	Celliac Disease	2	2	1057	N/A	Yes	Yes	No
31	[69]	VDR	Fractures	3	9	23309	Logistic regression	No	Yes	No
32	[96]	G72/G30	Schizophrenia	2	2	1541	N/A	Yes	Yes	Yes
33	[68]	VEGF	ALS	3	4	1912	Logistic regression	Yes	Yes	Yes
34	[126]	BANK1	Rheumatoid Arthritis	3	4	4445	1 vs. others	No	Yes	Yes
35	[67]	CYP19A1	Endometrial Cancer	2	10	13283	Logistic regression	No	Yes	Yes
36	[127]	CRP	T2DM	3	3	11876	N/A	No	No	Yes
37	[66]	8q24	Colorectal Adenoma	4	3	5385	Logistic regression	No	Yes	Yes
38	[128]	CYP1A1	Lung Cancer	2	13	2151	Pooled	No	Yes	Yes
39	[91]	TNFA	Prostate Cancer	5	2	4881	Pooled	Yes	Yes	No
40	[90]	PTGS2	Prostate Cancer	4	2	4881	Pooled	Yes	Yes	No
41	[129]	AR	Endometrial Cancer	5	2	1424	Pooled	No	Yes	No
42	[130]	MGMT	Head and Neck Cancer	2	3	1347	Pooled	No	Yes	No
43	[131]	SNCA	Parkinson Disease	2	11	5344	1 vs. other	No	Yes	Yes

We list the reference, the gene name, the disease, the number of SNPs included in the haplotypes, the number of studies, the total sample size, the method of analysis (N/A: not available), the availability of data, whether the data was collected in a collaborative setting and whether the study reported significant results.

The average number of polymorphisms included in the haplotypes was 3.19 (SD = 1.37, median = 3, range from 2 to 7), whereas the sample size was 5,017.81 (SD = 4,703.24, median = 3,004, range from 348 to 23,309). The average number of included studies was 5.14 (SD = 3.06, median = 4, range from 2 to 13). Twenty seven studies (62.79%) were conducted in a collaborative setting, whereas sixteen (37.21%) were performed using data derived from the literature. Twenty seven of the meta-analyses (62.79%) reported significant results and the majority (22 studies, 51.16%) were analysed under the "1 vs. others" approach using standard summary based meta-analysis techniques (with fixed or random effects), 11 studies (25.58%) were analysed by pooling the data inappropriately, 6 studies (13.95%) did not report the method or did not perform pooling at all and 4 analyses (9.30%) were performed using a fixed effects logistic regression model. Only 13 studies (30.23%) reported the complete data that suffice for the analysis to be replicated (Table 2 and 3).

There was only some weak evidence where collaborative meta-analyses contained larger number of studies compared to literature-based ones (5.67 vs. 4.25), larger sample size (5,651 vs. 3,948) and produced significant results more frequently (66.67% vs. 56.25%). However, these differences did noreach statistical significance (p-values equal to 0.144, 0.256 and 0.506 respectively). The average number of included polymorphisms was also comparable (3.26 vs. 3.06, p-value = 0.654). The thirteen meta-analyses that reported complete data, did not differ significantly from the remaining ones in terms of the included studies (4.46 vs. 5.43, p-value = 0.345), the number of SNPs in the haplotypes (3.08 vs. 3.23, p-value = 0.735) and the proportion of significant findings (69.23% vs. 60%, p-value = 0.576). The proportion of collaborative analyses was higher, even though this difference did not reach statistical significance (76.92% vs. 56.57%, p-value = 0.216). There was however, moderate evidence that the total sample size included in the meta-analyses that reported complete data was smaller compared to the meta-analyses that did not (3,040.31 vs. 5,874.73, p-value = 0.069). We also compared the particular database against a database of 55 representative meta-analyses of genetic association studies of SNPs that was used previously in several empirical evaluations [85-89]. The mean sample size was approximately equal (5,017 vs. 4,829, p-value = 0.844), but the number of included studies was nearly halved in the meta-analyses of haplotypes (5.14 vs. 10.53, p-value < 10^-4^), whereas the proportion of meta-analyses with significant results was twice as large (62.8% vs. 27.27%, p-value = 0.0003).

The thirteen studies that reported the data necessary for the analysis to be replicated were subsequently used in order to apply the methods proposed in this work. We used all the methods described in the methods section except for the simpler approach of comparing 1 vs. the others haplotypes, i.e. Eq.(5). The results are reported in Table 3, where we list the p-values for the tests for the overall association (**β **= **0**). For the fixed effects IPD methods we additionally report the p-value of the overall test for the heterogeneity (**γ = 0**). Concerning the results obtained using the IPD methods, we report only the ones obtained from the logistic regression method of Eq. (22) using the parameterization of Eq. (37) which is easier to be fitted, even though the multinomial logistic regression and the Poisson regression method would yield similar results. As expected, when the heterogeneity is low (in 8 out of the 13 studies), the random effects methods coincide with their fixed effects counterparts. In general, the methods that use summary data yield slightly different estimates for the ORs compared to the methods that use IPD, when there were rare haplotypes (i.e. small counts) or when the total number of subjects was low (data not shown). In 2 out of the 13 studies the estimates for the multivariate Wald tests for the overall association (**β = 0**) produce marginally different results compared to the univariate ones.

**Table 3:** The results obtained using the methods described in this work on the 13 studies that reported complete data that suffice for the analysis to be replicated

ID/[reference]	Gene/Locus	Disease/Outcome	SNPs in haplotype	Number of studies	Significant results	Fixed effects			Random effects	
						β = 0 (summary data)	β = 0 (IPD)	γ = 0 (IPD)	β = 0 (summary data)	β = 0 (IPD)
2/[98]	ITGAV	Rheumatoid Arthritis	3	3	Yes^$$^	0.2506	0.2489	0.1564	0.3288	0.3851
5/[99]	CX3CR1	CAD	2	6	Yes^$^	0.0834*	0.0677*	0.6263	0.0883*	0.1031*
16/[94]	CTLA4	Graves Disease	2	10	Yes	<0.0001	<0.0001	0.0371	<0.0001	<0.0001
17/[94]	CTLA4	Hashimoto Thyroiditis	2	5	Yes	0.0011	0.0010	<0.0001	0.0044	0.0072
20/[97]	CAPN10	T2DM	3	11	Yes^$$^	0.1152	0.1036	0.6145	0.2243	0.1655
21/[93]	ADAM33	Asthma	5	3	No	0.6209	0.5508	0.4697	0.6134	0.5503
26/[78]	VDR	Osteoporosis	3	4	Yes^$$^	0.1458	0.3051	<0.0001	0.1480	0.5781
27/[95]	ACE	Alzheimer's Disease	3	4	Yes	0.0193	0.0218	0.8906	0.0193	0.0223
30/[92]	FcgammaR	Celliac Disease	2	2	No	0.7331	0.7335	0.9502	0.7331	0.7336
32/[96]	G72/G30	Schizophrenia	2	2	Yes^$$^	0.7790	0.7757	0.0001	0.5750	0.6719
33/[68]	VEGF	ALS	3	4	Yes^$^	0.0437*	0.0414	0.0691	0.0716	0.0455*
39/[91]	TNFA	Prostate Cancer	5	2	No	0.2531	0.2515	0.6185	0.2867	0.2511
40/[90]	PTGS2	Prostate Cancer	4	2	No	0.3560	0.3550	0.2087	0.6573	0.4829

For either fixed or random effects methods, we list the p-values for the tests for the overall association (**β = 0**) using the summary data based methods and the IPD methods. The results for the IPD methods were obtained from the logistic regression method even though the multinomial logistic regression and the Poisson regression method yield nearly identical results. For the fixed effects IPD methods we also list the p-value of overall test for the heterogeneity (**γ = 0**).

(*): The significance of the multivariate Wald test (**β = 0**) contradicts univariate one (*β*_*j *_= 0).

(^$^): The initially claimed statistically significant results are contradicted by either the multivariate or univariate Wald tests (random effects).

(^$$^): The initially claimed statistically significant results are contradicted by both the multivariate and univariate Wald tests (random effects).

The subsequent re-analysis and the contrasting with the initial reports yielded some important findings. Concerning the four studies that initially reported no significant association [90-93], the methods presented in this work largely support the initial conclusions. Three of the nine studies (33.33%) that reported statistically significant results [94,95] yielded results that are in complete agreement with the initial reports (the meta-analysis of Kavvoura and co-workers reported results for two outcomes and it was counted twice). The most important finding, however, was the observation that 4 out of the 9 studies (44.44%) [78,96-98], yielded results that contradict the initial reports. Two additional studies [68,99] produced marginally significant results as judged by the disagreement between the multivariate and univariate Wald tests (Table 3).

The reasons for these discrepancies deserve further investigation. For instance, in the collaborative meta-analysis for the association of CAPN10 haplotypes with Type 2 Diabetes mellitus [97], the authors report a marginally significant OR of 1.09 (1.00, 1.18) for the "1-2-1" haplotype and similar results for two haplogenotypes that include this haplotype. Similar results were previously reported in a literature-based meta-analysis [100]. However, these estimates have been derived using the "1 vs. others" approach, which although more powerful, it is known to suffer from increase type I error rate; thus it seems that these estimates are the result of a multiple testing procedure. For the meta-analysis concerning the association of ITGAV haplotypes with Rheumatoid Arthritis [98], as well as the association of G30/G72 haplotypes with schizophrenia [96], the authors did not explicitly state how the pooling of estimates was performed, but the methods presented in this work suggest clearly that there is not enough evidence supporting the claimed associations. Finally, in the case of the meta-analysis for the association of VDR polymorphisms with osteoporosis, in which the authors claimed to use a log-linear model [78], the initially drawn conclusions are not supported. It seems that the authors did not use a correctly specified model that contains all the main effects as well as all the two-way interactions (i.e. the "*no three-factor interaction model*"). This probably resulted in performing a meta-analysis essentially without stratifying by study. Given that in the particular dataset the heterogeneity is large, it is of no surprise that the originally drawn conclusions are compromised after the re-analysis, which strongly indicates that there is no evidence to support a significant association. Concerning the two datasets for which we observed disagreement between the multivariate and univariate Wald tests, i.e. the association of CX3CR1 haplotypes with CAD [99] and the association of VEGF haplotypes with ALS [68], there were different reasons for the discrepancies. In the meta-analysis of CX3CR1 haplotypes (which was originally performed using the "1 vs. others" approach) the small discrepancies could be attributed to the marginal statistical significance (p-values = 0.06-0.09) and the existence of a rare haplotype. In the case of the VEGF meta-analysis, the authors initially used a fixed-effects logistic regression model analogous to Eq. (17); however, the moderate heterogeneity produced slight discrepancies in the results of the multivariate Wald test under the random effects model (Table 3).

## Discussion

Although the studies reporting haplotypes comprise a small fraction of genetic association studies, their number is increasingly growing and so there is a need for developing formal methods for combining them in a meta-analysis. In this work, a comprehensive framework for the meta-analysis of haplotype association studies was presented and an empirical evaluation has been performed for the first time in the literature.

The methods proposed in this work are extending previous works in meta-analysis of genetic association studies [12,16] in order to handle the multiple haplotypes. These works in turn, are based on the previously described large corpus of methods for multivariate meta-analysis [33,36,37,62,101-103]. We proposed summary-data based methods as well as methods for IPD. Although the former are very easily implemented, the latter provide some very useful insights. By viewing the meta-analysis data as a 2 × *r *× *k *contingency table [45] allowed developing methods based on logistic regression, multinomial logistic regression and Poisson regression. Although logistic regression methods have long being used for meta-analysis of IPD [33,36,37], multinomial logistic regression has only being used for meta-analysis of genetic association studies under the retrospective likelihood [12,80]. Most importantly, Poisson regression models have been used in entirely different contexts, such as survival analysis [104] and meta-analysis of follow-up studies with varying duration [105]. Thus, an important advancement of this work is the extension of the commonly used approach for analyzing haplotype data [43,44] in the meta-analysis setting, describing appropriately specified models and presenting them in a unified framework (i.e. the contingency table analysis).

The empirical evaluation of the published literature suggests that studies reporting meta-analysis of haplotypes did not systematically differ from the meta-analyses of genetic association using SNPs in terms of the average sample size, but contain approximately half of the included studies and produce significant results twice more often. The meta-analyses that reported the complete data did not significantly differ from the remaining studies in terms of the included studies, the number of SNPs included in the haplotypes, the proportion of significant findings or the proportion of collaborative analyses. There was however, moderate evidence that the total sample size included in the meta-analyses that reported complete data, was smaller compared to the meta-analyses that did not.

The application of the methods proposed in this work in studies that reported the complete data, made clear that approximately half of the significant findings are attributable to the method of analysis used by the primary authors and suffer from an inflated type I error rate. Indeed, for the four out of the nine studies that reported significant results, these were clearly refuted by the multivariate methodology. Three of these studies used the 1 vs. other approach, which although more powerful, is known to suffer from increased type I error rate [61], whereas the results of the fourth study were based on a misspecified log-linear model. Two additional studies produced marginally insignificant results (i.e. the multivariate Wald test contradicted the univariate one), mainly due to the existence of rare haplotypes or heterogeneity that has not been accounted for in the initial analysis.

All the models presented here assume that the haplotypes are directly observed. However, as we have already discussed, the haplotypes are usually inferred and thus, treating them as known quantities may be problematic [30]. The general framework presented in this work can be easily extended in order to account for this uncertainty, simply by weighting the inferred haplotypes by their probability [49,50]. However, this will probably be problematic in many real life applications, except when dealing with a collaborative analysis, since a meta-analyst will rarely have access to individual genotype data in order to use them to estimate the haplotypes and their posterior probabilities. If combined genotypes are available for all studies, the meta-analyst may try to re-construct the haplotypes with a method of his/her choice and perform the analysis using the posterior probabilities as weights. Moreover, if individual genotype data is available (from the literature or in a collaborative setting), the framework can be extended to allow the haplotype risk to follow models of inheritance other than the multiplicative one (i.e. estimating the risk of haplogenotypes), or to include patient-level covariates.

The methods proposed in this work, clearly outperform the traditional naïve method of meta-analysis of haplotypes, which simply consists of contrasting each haplotype against the remaining ones. This is expected to be more profound, especially as the number of possible haplotypes increases, increasing also the type I error rate due to multiple comparisons [59,60]. Collapsing the haplotypes and performing a univariate analysis, may potentially be more powerful in several situations [61]. However, in genetic association studies, even though we are interested in small genetic effects we are also concerned about the probability of false findings [106,107]. Thus, the multivariate methodology seems to be a reliable alternative.

## Conclusions

We presented multivariate methods that use summary-based data as well as methods that use binary and count data in a generalized linear mixed model framework (logistic regression, multinomial regression and Poisson regression). The methods presented here are easily implemented using standard software such as Stata, R or SAS making them easy to be applied even by non- experts. In the Additional file 1, Stata code for fitting the models described in this work is given and we expect that these methods will be widely used in the future.

## Authors' contributions

PGB conceived the study, performed the analyses and wrote the manuscript.

## Supplementary Material

Additional file 1 **Stata code for fitting the methods described in the manuscript**. The commands should be run within a Stata do-file. Click here for file
