# Supplementary material for: Meta-analysis of haplotype-association studies: comparison of methods and empirical evaluation of the literature
Source: BMC Genet. 2011 Jan 19;12:8. doi: 10.1186/1471-2156-12-8 (PMC3087509; doi:10.1186/1471-2156-12-8)
Supplement: Additional file 1 — Stata code for fitting the methods described in the manuscript. The commands should be run within a Stata do-file. [file 1471-2156-12-8-S1.DOC]

**Appendix**

** The data are taken from the meta-analysis for the association of

** CTLA4 haplotypes and Graves Disease (Kavvoura et al, 2007)

** The commands should be run within a do-file

clear

input study a1 a0 b1 b0 c1 c0 d1 d0

1 162 111 23 36 12 22 81 141

2 149 432 2 5 26 92 39 159

3 421 221 1 2 71 39 111 96

4 76 117 7 7 45 48 68 100

5 293 256 1 4 126 122 182 236

6 336 297 6 21 43 58 85 102

7 394 113 0 0 64 17 116 60

8 21 59 0 4 15 32 52 105

9 222 234 2 0 28 35 44 73

10 589 625 0 1 262 269 493 793

end

** Calculation of the log Odds Ratio

** for the 1 vs. others approach (haplotype d)

** Equations (3.1) -(3.4)

gen nod1= a1+ b1+ c1

gen nod0= a0+ b0+ c0

gen logorh=log((d1/nod1)/(d0/nod0))

gen seh=sqrt(1/d1+1/d0+1/nod1+1/nod0)

metan logorh seh,eform randomi

** calculation of the log Odds Ratios for the multivariate meta-analysis

** Equation (3.6)

gen h1=log(( b1/ a1)/( b0/a0))

gen h2=log(( c1/ a1)/( c0/a0))

gen h3=log(( d1/ a1)/( d0/a0))

** continuity correction

replace h1=log(( (b1+0.5)/(a1+0.5))/( (b0+0.5)/(a0+0.5))) if b1==0 |b0==0

replace h2=log(( (c1+0.5)/(a1+0.5))/( (c0+0.5)/(a0+0.5))) if c1==0 |c0==0

replace h3=log(( (d1+0.5)/(a1+0.5))/( (d0+0.5)/(a0+0.5))) if d1==0 |d0==0

** calculation of the variances

** Equation (3.7)

gen v11=1/a0 +1/b0+1/a1 +1/b1

gen v22=1/a0 +1/c0+1/a1 +1/c1

gen v33=1/a0 +1/d0+1/a1 +1/d1

** continuity correction

replace v11=1/(a0+0.5) +1/(b0+0.5)+1/(a1+0.5) +1/(b1+0.5) if b1==0 |b0==0

replace v22=1/(a0+0.5) +1/(c0+0.5)+1/(a1+0.5) +1/(c1+0.5) if c1==0 |c0==0

replace v33=1/(a0+0.5) +1/(d0+0.5)+1/(a1+0.5) +1/(d1+0.5) if d1==0 |d0==0

** calculation of the covariances

** Equation (3.11)

gen v12 = 1/a0 +1/a1

gen v13 = 1/a0 +1/a1

gen v23 = 1/a0 +1/a1

** fitting model of Equation (3.8)

mvmeta h v,vars(h1 h2 h3) mm

** data re-arrangement

reshape long a b c d, i(study) j(case)

gen id = _n

rename a count1

rename b count2

rename c count3

rename d count4

reshape long count, i(id) j(hap)

gen wt1=count

xi i.study i.hap i.study*i.hap i.study*case i.hap*case

foreach var of varlist _IstuXhap_2_2- _IstuXhap_10_4{

quietly generate _IcaX`var'=case*`var'

}

** fitting the fixed-effects logistic regression model

** Equation (3.12)

logit case _Ihap_* _Istudy_* [fw=count]

** overal score test for association

testparm _Ihap_*

** fitting the fixed-effects logistic regression model with interaction

** Equation (3.13)

logit case _Ihap_* _Istudy_* _IstuXhap_* [fw=count]

** test for heterogeneity

testparm _IstuXhap_*

** fitting the fixed-effects multinomial logistic regression model

** Equation (3.20) -(3.21)

mlogit hap _Istudy_* case [fw=count],b(1)

** overal score test for association

testparm case

** fitting the fixed-effects multinomial logistic regression model

** with interaction, Equation (3.22)

mlogit hap _Istudy_* case _IstuXcase*[fw=count],b(1)

** test for heterogeneity

testparm _IstuXcase_*

** fitting the fixed-effects Poisson logistic regression model

** Equation (3.24)

poisson count _Istudy_* _Ihap_* case _IstuXhap_* /*

*/_IstuXcase_* _IhapXcase_*

** overal score test for association

testparm _IhapXcase_*

** fitting the fixed-effects Poisson logistic regression model

** with 3-way interaction

** Equation (3.25)

poisson count _Istudy_* _Ihap_* case _IstuXhap_* /*

*/_IstuXcase_* _IhapXcase_* _IcaX*

** test for heterogeneity

testparm _IcaX*

** fitting the random-effects logistic regression model

** Equation (3.17)

** we use the covariance matrix parametrization of Equation (3.32)

eq slope: _Ihap_2 _Ihap_3 _Ihap_4

gllamm case _Ihap_2 _Ihap_3 _Ihap_4 _Istudy_*, fam(binom) link(logit) /*

*/ i(study ) nrf(1) eqs(slope) w(wt) adapt nip(8)

** overal score test for association

test [case]_Ihap_2 [case]_Ihap_3 [case]_Ihap_4

** fitting the random-effects logistic regression model

** Equation (3.17)

** using the covariance matrix parametrization of Equation (3.19)

** note, that this could be very time-demanding

eq slope2: _Ihap_2

eq slope3: _Ihap_3

eq slope4: _Ihap_4

gllamm case _Ihap_2 _Ihap_3 _Ihap_4 _Istudy_*, fam(binom) link(logit) /*

*/ i(study ) nrf(3) eqs(slope2 slope3 slope4) w(wt) adapt nip(8)

** overal score test for association

test [case]_Ihap_2 [case]_Ihap_3 [case]_Ihap_4
